# Identifying the scope of ethical challenges caused by the Ebola epidemic 2014-2016 in West Africa: a qualitative study

**DOI:** 10.1186/s13010-022-00128-y

**Published:** 2022-12-28

**Authors:** Saskia Wilhelmy, Regina Müller, Dominik Gross

**Affiliations:** 1 Institute for History, Theory and Ethics of Medicine, Medical Faculty, RWTH Aachen University, Wendlingweg 2, 52074 Aachen, Germany; 2 Institute of Philosophy, University of Bremen, Enrique-Schmidt-Str. 7, 28359 Bremen, Germany

**Keywords:** Epidemic, Ebola, Bioethics, Ethical implications, Media

## Abstract

**Background:**

The West African Ebola virus epidemic from 2014 to 2016 is unprecedented in its scale, surpassing all previous and subsequent Ebola outbreaks since 1976. This epidemic provoked a humanitarian emergency that extended to different spheres of life, making visible ethical challenges in addition to medical, economic, and social ones. The present article aims to identify and differentiate the scope of ethical issues associated with the Ebola epidemic.

**Methods:**

An online media analysis was performed on articles published from March 2014 to September 2015 in ten preselected academic journals (scientific press) and two online newspapers (lay press). Two methodological approaches were combined: a systematic literature search and a qualitative content analysis. An additional keyword search was conducted on the PubMed database for the period after the end of the Ebola epidemic (2016-2020) to obtain an overview of research dealing with medical ethics due to the epidemic and to compare these results with the identified ethical challenges.

**Results:**

A total of 389 articles dealing with the subject fields “Ebola epidemic” and “ethics” were researched. For qualitative content analysis, the time span with the highest article density was selected and a total of 64 articles were included (15 scientific articles, 49 popular articles). Five core ethical challenges of the Ebola epidemic emerged: 1. Responsibility and Accountability, 2. Spillover Effects, 3. Research and Development, 4. Health Communication, and 5. Resource Allocation. Articles in academic journals were dominated by the discussion of normative aspects in the area of “research and development”, while newspaper articles focused on aspects of “responsibility and accountability”.

**Conclusion:**

An ethical discussion of the Ebola epidemic requires an examination of as many of the ethical dimensions involved as possible. The presented investigation of the two types of media with regard to the Ebola epidemic offers this possibility of a more comprehensive insight into this diversity as a basis for ethical discussions.

## Background

On 17 July 2019, the World Health Organization (WHO) declared for the Democratic Republic of Congo (DRC) (formerly Zaire) that the conditions for the declaration of a Public Health Emergency of International Concern were met, as the Ebola virus disease (EVD) has been prevalent in the republic for one year [1–3]. In the history of Ebola outbreaks, this is the second most deadly outbreak since the first appearance of the disease and still poses a threat to public health [4]. The situation in the DRC in Central Africa is reminiscent of the health emergency declared five years ago in West Africa, on 8 August 2014, due to a rampant Ebola epidemic [5]. Although EVD has been known since the 1970s, the epidemic in 2014 became the focus of global attention for the first time, as the outbreak exceeded all known dimensions, causing 11,310 deaths in several West African countries (and a total of 28,616 reported cases) [6]. Several factors led to this unprecedented outbreak, such as insufficient public health infrastructure, difficulty aligning infection control with some cultural practices and problems in crisis management [7]. These factors can be partly attributed to poor or non-targeted global support, so that the aspect of neglect regarding the outbreak plays a crucial role. Regarding the lack of research and development of vaccines and antivirals in the affected regions, structural adjustments (e.g. regarding ethics, fair access to interventions, sharing of research results etc.) as well as political and armed conflicts also play a role [8]. Indeed, during the outbreak (early 2014) or during the spread and containment (early 2016) of the Ebola epidemic, no approved or adequately tested preventive or therapeutic agent was available [9]. Although there have been previous projects to research suitable vaccines or antivirals, such as for Ebola (e.g. project Bioshield, https://www.medicalcountermeasures.gov/barda/cbrn/project-bioshield-overview), it was not until the end of 2016 that a Canadian scientific research group led by the WHO published the results of a study on a vaccine (rVSV-ZEBOV) against the Ebola virus; this vaccine promised to be highly effective [10] and was thus the first suitable vaccine in forty years of Ebola’s existence. The Ebola outbreak led to an intensification and expansion of existing research on the virus [11], but a suitable and approved treatment, such as a drug or vaccine, was lacking during the epidemic in 2014-2016. Therefore, activities to contain the virus focused on hygiene, protection, quarantine and symptomatic interventions as well as supportive care to reduce the death rate [12].

There are several reasons for the neglect in the field of Ebola research, but the main reason is probably the low level of concern regarding the Ebola virus in high-income countries: On the one hand, there is no civil or economic pressure to respond to an immediate threat, and on the other hand, there are no profitable incentives for the pharmaceutical industry.

This perspective changed during the epidemic due to the immense number of people infected and the potential international threat posed by the virus. In summary, this constellation provoked ethical dilemmas due to the simultaneous strong pressure to act and the lack of suitable means for action: “Responding effectively to an outbreak of disease often requires routine processes to be set aside in favor of unconventional approaches. Consequently, an emergency response situation usually generates ethical dilemmas.” [13].

As the responsible coordination and action agency for international public health, the WHO tried to bring the dramatic and threatening situation in West Africa under control. Independent experts convened by the WHO finally decided that the use of experimental agents in the context of the epidemic was justified, provided that the following criteria were met: “[…] transparency about all aspects of care, so that the maximum information is obtained about the effects of the interventions, fairness, promotion of cosmopolitan solidarity, informed consent, freedom of choice, confidentiality, respect for the person, preservation of dignity, involvement of the community and risk–benefit assessment.” [14]. This decision led to a controversial discourse on the use of experimental agents in the Ebola epidemic, ranging from arguments such as unpredictable health problems and long-term consequences of their use, to follow-up issues such as the fair distribution of drugs to needy groups of people. In addition to this research-ethical focus, various other ethical questions were discussed.

In the course of the Ebola epidemic 2014-2016, several research studies focused on epidemiological, biological or medical aspects of the virus and the epidemic. However, the systematic identification of bioethical aspects to determine the extent of the ethical challenges of the epidemic continued to be a research desideratum. Against this background, an inductive methodological approach was chosen that allowed for an exploratory identification of the spectrum of ethical challenges within the textual base.

Aim of the present investigation is to document and analyze ethical challenges raised during the Ebola epidemic 2014-2016 on the basis of articles in academic journals and newspapers. The challenges identified were then considered in relation to the two different types of media and post-epidemic ethical research on the 2014-2016 Ebola outbreak.

## Methods

The ethical challenges of the epidemic in the media were analyzed in two successive phases: The first (quantitative) step was to create a database, while the second (qualitative) step served to gain a more detailed insight into the ethical challenges. A systematic literature search was conducted primarily on bibliographic databases. Subsequently, a qualitative content analysis [15] was performed, focusing on the time span with the highest article density over the study period. Data on thematically relevant articles in preselected (national and international) academic journals and in newspapers were collected and analyzed. The overall study covered the period between 1 March 2014 (official announcement of the Ebola outbreak by the WHO, 23 March 2014) and 1 September 2015 (start of EVD research project). An additional keyword search was conducted on the PubMed database for the period after the end of the Ebola epidemic (2016-2020) to obtain an overview of research dealing with medical ethics due to the epidemic and to compare these results with our identified ethical challenges.

### Literature search

A total of 24 academic journals (with open access; Bioethica Forum, Bulletin zur Arzneimittelsicherheit, Bundesgesundheitsblatt Gesundheitsforschung Gesundheitsschutz, Bull World Health Organ, Dtsch Arztebl, Dtsch Med Wochenschr, Epid Bull, EthikJournal, Ethik Med, GMS MBIE, GMS Z Med Ausbild, JAMA, J Bioeth Inq, J Epidemiol Community Health, J Law Med Ethics, J Med Ethics, J Public Health (Oxf), Lancet, MedR, Nature, Public Health Forum, Science, ZfmE, ZfMER) from various disciplines (medicine, medical ethics, public health, infectiology, epidemiology, tropical medicine and natural science) were selected. In terms of content, the journals were examined to determine whether they (1) focus on Ebola (2014-2015) and (2) discuss ethical challenges. Of these 24 journals, 16 had Ebola related content, but only 10 of them also addressed ethical aspects. Accordingly, the database consisted of 10 academic journals (Bull World Health Organ, J Bioeth Inq, J Law Med Ethics, J Med Ethics, JAMA, Lancet, Nature, Science, Dtsch Arztebl, MedR), eight of which were from the English-speaking world and two from the German-speaking area. On this basis the scientific discourse was explored (scientific press).

In addition to the academic journals, two daily newspapers (Washington Post and Welt Online) were selected (one from the English-speaking world and one from the German-speaking area). Selection criteria were the treatment of social issues (especially Ebola), high impact and reach, and online availability. Here the aim was to explore the public discourse (lay press).

The first step was a systematic keyword search focusing on Ebola terms, which resulted in a total of 888 articles from the 10 academic journals and the two daily newspapers. The objective was to obtain a comprehensive overview of the subject field “Ebola epidemic” with the priority on completeness. Explicit keywords combined into three categories were used to identify relevant articles. The first category was “Ebola” (e.g. specified keywords: Ebola virus, Ebola fever; general keywords: hemorrhagic fever, infectious disease), the second category related to the Ebola theme was “West Africa” (e.g. specified keywords: Sierra Leona, Guinea; general keyword: Africa), and the third category was “ethics” (e.g. specified keywords: applied ethics, medicine ethics; general keywords: moral (theory), philosophy).

**Fig. 1:**
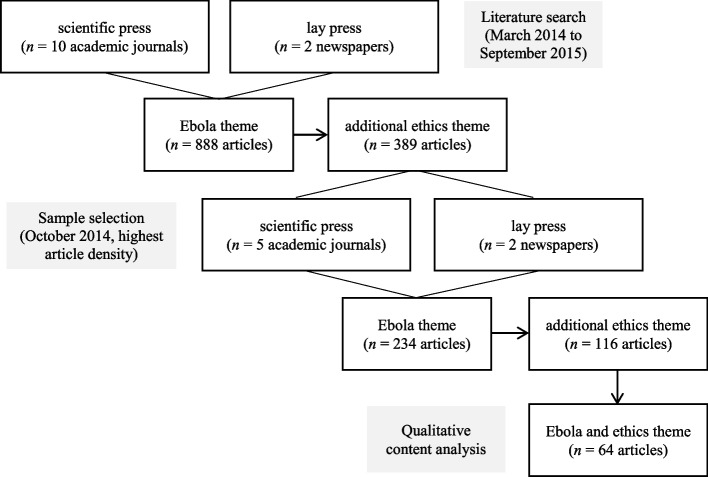
Data collection. Results of the literature search (2014-2015)

The online database LexisNexis was used as a research platform to identify articles in the two daily newspapers. The online search catalogs of the academic journals were consulted to find relevant scientific articles. In addition to the technology-based search, a manual review of the articles was performed using keywords. In a second step, a sample size of 389 articles on Ebola was generated with an additional ethical reference for the entire study period (see Fig. 1); these articles were fixed in a database.

### Sample selection (theory of the dynamics of media attention)

The theory of the dynamics of media attention [16] was used to select a sample of articles from the investigation period, which was used for the qualitative content analysis. Public communication processes within the media coverage can follow certain dynamics, i.e. there are idiosyncratic cycles during the reporting period in which topics range from a very strong media presence to complete disappearance from the media; this specific course is called a “topic career”. The theme and attention cycle is a reference model to illustrate topic careers. This model shows that the trend of a topic within the media follows a certain pattern. Accordingly, a topic career passes four phases: The latency period, the thematic breakthrough, the “coming into fashion” theme and the fatigue phase [16].

In the latency period the topic is only of interest to initiates, i.e. it is not represented among the general public but rather to persons who are directly affected or thematically involved and who specifically deal with the topic (niche topic). When there is increased reporting (i.e. not only by specialized persons) over the course of time, this is referred to as a “thematic breakthrough”. This phase is characterized by an increased frequency of articles. The topic can then “come into fashion” over the further course of time, i.e. the topic takes precedence in reporting, and article density grows strongly until a maximum point is reached. When a maximum has been exceeded, reporting on the subject returns to a low level (reasons could be, e.g. overloading of subjects, exhausted information resources or even new topic careers which already replace existing ones).

The sample used for qualitative content analysis in our study consists of articles published in the “come into fashion” phase. In this phase the number of articles is highest, at the same time a high density of different focal points on the topic “Ebola epidemic” can be assumed. The articles were read and examined according to three explicit exclusion criteria: thematic distance (e.g. epidemiological or clinical contents on Ebola only) and wrong text type (only descriptive reporting and no discussion).

### Qualitative content analysis

Qualitative content analysis [15] was used to analyze the articles. The contents of the articles were systematically scrutinized in terms of inductive category development. Unlike a deductive approach, in which the articles are examined with regard to specific pre-determined ethical issues, an inductive methodological approach enables an explorative identification of ethical challenges in the textual basis, from which categories can be derived that demonstrate the ethical spectrum of the challenges. A detailed workflow was used to investigate the database in order to comply with the research objective (identification of ethical challenges of the Ebola epidemic) and to adhere to quality criteria of empirical measurement (e.g. transparency, intersubjectivity, generalizability). The articles were examined completely and independently by two coders, who monitored each other during the work process and resolved differences by consensus.

In a first step, the contents were determined by coding all significant text passages. Within this content classification, a broad understanding of ethics was presupposed. There are different understandings of how to recognize an ethical problem as such; even at the conceptual level, there are different interpretations of what is meant by this or how to name it properly [17]. In accordance with an approach to identify ethical problems in literature [18] two guiding questions were developed: 1) Is an ethical problem addressed in the article? 2) Is this ethical problem explicitly mentioned or is it implicitly perceived by the coder?

Furthermore, it was investigated whether the content of the articles described either an ethical deficit (type I) or an ethical uncertainty (type II). Type I is subsumed by an obvious ethical deficit or ethical misconduct, i.e. situations where established ethical rules/values have been violated; e.g. involving individuals in clinical trials without informing them and obtaining their consent. Type II is characterized by uncertainty about the ethically appropriate course of action. This involves situations in which it is unclear which practices are right, as there are both arguments for and against them from an ethical point of view – e.g. the inclusion of children in clinical trials [18]. The article contents were abstracted, i.e. the core contents of the articles were retained and grouped into categories. Consequently, an article could be assigned to several categories. Afterwards, the abstracted results were double-checked on the database and, if necessary, changed into a higher level of abstraction.

The category system developed was reviewed after approximately 20% of the database had been processed. In a final step, the remaining articles were subsumed under existing categories or, if necessary, new categories were established accordingly.

## Results

**Fig. 2:**
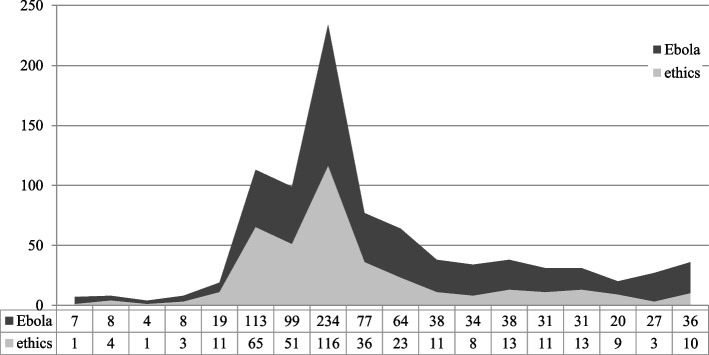
Articles on Ebola and ethics. Number of articles on Ebola (*n* = 888) and additional ethical aspects (*n* = 389) during the entire study period (2014-2015)

The results of the literature search show that the “Ebola epidemic” topic career follows the pattern of the media dynamics model. The sample size of the period “come into fashion” comprised 234 articles with Ebola themes and was reduced to 116 articles with additional ethical content (see Fig. 2). As a final result (after application of the exclusion criteria), the sample size from October was reduced to a total of 64 articles, which were then used as basis for qualitative content analysis.

The results of the qualitative content analysis provide insights on the range of ethical challenges during the Ebola epidemic 2014-2016. It was possible to establish a total of five categories from the 64 articles of October 2014: 1. *Responsibility and Accountability*, 2. *Spillover Effects*, 3. *Research and Development*, 4. *Health Communication*, 5. *Resource Allocation*. The majority of these main categories were divided into subcategories (see Table 1).

1. **Responsibility and Accountability**

**Table 1:** Ethical challenges: five main categories including subdivisions

	total (*n*)	scientific articles (*n*)	popular articles (*n*)
Categories/subdivisions			
**1. Responsibility and Accountability**	**45**	**7**	**38**
Negative obligation of assistance	27	2	25
Positive obligation of assistance	18	5	13
**2. Spillover Effects**	**29**	**8**	**21**
Fear	10	3	7
Stigmatization	10	2	8
Economic consequences	4	1	3
Neglect of other diseases	3	2	1
Humor	2	0	2
**3. Research and Development**	**14**	**10**	**4**
Implementation	9	7	2
Financing	5	3	2
**4. Health Communication**	**11**	**4**	**7**
Scaremongering and misinformation	8	3	5
Public interest vs. personal rights	3	1	2
**5. Resource Allocation**	**4**	**0**	**4**

Identified and encoded categories (October 2014) and subcategories ordered by number of articles (*n*) as they appear in the two media types (academic journals/scientific articles; newspapers/popular articles)

The most frequently identified category in the database is *Responsibility and Accountability* (in total, *n* = 45 articles). It contains discourse fragments which formulate an obligation between a person or group of persons (subject) towards another person or group of persons (object). Both reference points (subject and object) represent individuals (e.g. a nurse), institutions (e.g. the WHO) or societies (e.g. the country Sierra Leone). This concept of obligation is divided into two types (positive and negative obligation of assistance), which further specify the subject-object-relations. The aspect of positive obligation of assistance comprises statements which demand improvement to a situation. This requirement is usually found in the form of an appeal or a direct call for action as well as in the form of criticism of an omission of assistance (e.g. late or insufficient assistance: “[…] societies have an obligation to help people affected by Ebola […]. Virtually all high-income countries are thus in a position to effectively help curb the Ebola epidemic, without sacrificing much of importance. The moral obligation of humanitarian assistance requires doing so.” [19]; “The WHO has failed in the fight against Ebola. It declared a state of emergency after five months, acted too late and was badly prepared, critics say.” [20]; translated into English).

In contrast, the concept of negative obligation of assistance generally refers to the prevention or limitation of harm. Statements placed under this concept describe conditions which should not be allowed to worsen, i.e. persons (or groups) should be protected from further harm or should not be exposed to the risk of harm. What is meant here are (mostly preventive) security measures with the aim of protecting others from infection with the Ebola virus. It comprises characteristics like (self-)quarantine measures (e.g.: “There’s a man on the subway through New York, even though he’s infected with Ebola. And though he should know that. Because the man is a doctor and is just coming back from an emergency in a disease area. […] He acts against his best knowledge and his moral impulse as a helper, because he does not want to endanger his own danger of death. In this example, the idea of indivisible reason and humanity is in danger. For there the survival of the individual becomes a threat to all.” [21]; translated into English), safety and security measures relating to travel and transport (e.g.: “There will be calls to restrict travel but that is not needed. What is needed now is to screen passengers getting on planes or boats to the U.S. for symptoms of Ebola.” [22]), medical staff (e.g.: “Nevertheless, it would be desirable for the authorities to take account of errors such as those in Madrid or even those in Dallas. [...] Why is the staff of the high-security stations of a clinic not better trained and checked?” [23]; translated into English) and (major) events (e.g.: “Because of sport, thousands of people come together in the world. For fear of the Ebola virus, precautions are taken — conflicts such as the footballer Bangoura from Guinea included.” [24]; translated into English). Statements that reflect negative obligations of assistance were more frequently found (*n* = 27 articles) than positive ones (*n* = 18 articles) in the database. Moreover, it can be summarized that statements on positive and negative obligation assistance (or the concept) were mainly found in newspapers (newspapers: *n* = 38 articles, academic journals: *n* = 7 articles).

2. **Spillover Effects**

A further main category, divided into six subcategories, refers to the spillover effects (*n* = 29 articles) of the Ebola epidemic. It comprises all statements that include the effects of the epidemic on different areas of life: (irrational) fear (*n* = 10 articles; e.g.: “The Ebola outbreak has pitted rational science against fear and superstition. We see this in Africa: the murder last month of eight people working to raise awareness of the disease close to the town of Nzerekore, in southeastern Guinea, is a tragic example.” [25]), stigmatization and discrimination (*n* = 10 articles; e.g.: “‘We avoid wearing our uniforms in public, because then all the others avoid us’, says nurse Mabel Saybay. Only at the hospital she changes her clothes. But the stigmatization takes on much worse forms: homeowners throw people out of their homes who work in hospitals or bury Ebola dead people [...]. To throw helpers out is ‘completely unacceptable and unpatriotic’, says deputy information minister Isaac Jackson.” [26]; translated into English), neglect of other diseases (*n* = 3 articles; e.g.: “As the Ebola death toll spirals into the thousands in West Africa, the outbreak could have a spillover effect on the region’s deadliest disease. The outbreak has virtually shut down malaria control efforts in Liberia, Guinea and Sierra Leone, raising fears that cases of the mosquito-borne illness may start rising — if they haven’t already.” [27]), economic consequences of the epidemic (*n* = 4 articles; e.g.: “At the end of September, the UN called on its member states to release a total of one billion dollars (800 million euros) for the fight against Ebola. Thus, from the neglected plague becomes suddenly a lucrative disease for the pharmaceutical industry.” [28]), and questions of humor (*n* = 2 articles; e.g.: “The New York Post has already called it this year’s ‘hot’ Halloween costume. But some in the field are saying it’s too soon to joke — as medical professionals are still fighting to stop the spread of the devastating disease that has killed more than 4,500 people in West Africa. ‘Normally I think that irony and humor is funny, but this thing with the costumes, is it really that funny? I mean, Ebola’s not even under control yet,’[…].” [29]).

3. **Research and Development**

The category *Research and Development* (*n* = 14 articles) summarizes ethical statements or questions that generally concern research on and the development of substances to treat Ebola, precise vaccines and medicines. These include arguments on funding (for example, the question of who should bear the cost of effective substances if no commercial benefits are expected), statements on the implementation of research and development (for example, questions concerning study design), as well as on the fair distribution of research results and effective substances. These subjects were in turn differentiated into the two sub-categories *Implementation* (e.g.: “Studies on efficacy in affected countries and more so in at-risk populations should not have a placebo or active control arm as this cannot be defended ethically […].” [30]) and *Financing* (e.g.: “Even if the pathogen has so far only occurred in remote regions. ‘The development of medicines and vaccines for the so-called neglected diseases must not depend on market considerations’, says Klenk.” [31]; translated into English). This main category was identified a total of 14 times as follows within the sample size of articles: academic journals (*n* = 10 articles) and newspapers (*n* = 4 articles). In the academic journals (namely: JAMA, Nature, Science, Dtsch Arztebl), a majority of 64% discusses the *Implementation* of research and development related to Ebola and only 36% reflect the category of *Funding*. By contrast, the distribution of the two subcategories in articles in the daily newspapers (The Washington Post, Welt Online) is balanced.

4. **Health Communication**

All statements in the database dealing with *Health Communication* (*n* = 11 articles) of the Ebola epidemic are included in this category. In addition, all discourse fragments denoting public interest in information about the epidemic and Ebola cases were classified here, i.e. transparency in public, and the protection of personality rights of affected persons (*n* = 3 articles; e.g.: “The public has a legitimate interest in knowing the places an infected person has frequented, for example, but there is a fine line between this and blatant voyeurism, invasion of privacy and sensationalism.” [32]). This category also includes statements criticizing the false and unreflected dissemination of information as well as anxiety, hysteria and panic triggered by public communication (*n* = 8 articles; e.g.: “Fear of Ebola as an uncontrollable disease is rampant worldwide. Every suspected case triggers breaking news on TV and online portals. One is reminded involuntarily of the media ‘overkill’ during the times of SARS, avian and new influenza.” [33]). Sources of the health communication considered here include the following mass media and further means of informing the public: classical mass media (newspapers, television, broadcasting and film), social media (micro-/blogs, content communities and social networks), literature (fiction) and public events.

5. **Resource Allocation**

The fifth major category, *Resource Allocation,* contains statements that are generally related to the distribution of resources. It was found a total of 4 articles. The topics were: distribution of drugs, treatment units and treatment costs (e.g.: “Cost issues should be addressed — who will pay for visitors coming here or any uninsured person who becomes infected?” [22]. This category was only identified in articles of the newspapers. It was not specified into further subcategories.

A core part of the analysis was to determine whether the ethical problems identified were explicitly mentioned in the articles or implicitly perceived by the coders. Mainly implicit ethical problems (89%) were identified and only a few explicit ones (11%) in the database. The majority of the explicit ethical challenges were found in articles of academic journals (*n* = 5); ten articles here indicated the existence of implicit ethical problems. In the newspapers, however, the majority (*n* = 47) of articles implicitly referred to ethical problems, whereas only two articles explicitly named ethical issues.

The categories were also distinguished into type I (ethical deficit) or type II (ethical uncertainty) (see Table 2). Only in the main categories *Research and Development* and *Resource Allocation* was type two predominant. In all other main categories, type one, i.e. ethical deficit prevailed (*Spillover Effects*: 90%; *Health Communication*: 82%).

**Table 2:** Ethical deficit vs. ethical uncertainty

	Categories	Ethical deficit (type 1), *n*	Ethical uncertainty (type 2), *n*	*n*, total
1.	Responsibility and Accountability	32	13	45
2.	Spillover Effects	26	3	29
3.	Research and Development	5	9	14
4.	Health Communication	9	2	11
5.	Resource Allocation	0	4	4

Distribution of ethical types (deficit, uncertainty) in the data set in relation to the identified categories (October 2014), ordered by number of articles (*n*)

## Discussion

The results of the current study show the multiple ethical challenges that were addressed during the Ebola epidemic in articles of the two selected media types. It becomes apparent that the challenges identified differ greatly between scientific (i.e. scientific articles) and lay (i.e. popular articles) press; the database shows that articles from academic journals were more frequently assigned to the category “Research and Development” (*n* = 10), while articles from the two newspapers were most frequently assigned to the category “Responsibility and Accountability” (*n* = 38). This is primarily due to the different characteristics and thematic focus of the two (online) media types, e.g. with regard to content, format, authors, recipients, organization, review and publication frequency [34]. This difference can also be seen in the way in which ethical problems are addressed: they are mostly mentioned explicitly in articles of academic journals and the majority of implicit ethical problems are mentioned in the newspapers. Thus, it can be assumed that the respective articles have certain genre characteristics depending on the assigned media type which thus gives the content a pre-selected perspective; this can depend on various factors, e.g. who the content of the articles is aimed at (readership) and thus what prior knowledge is assumed, what the thematic focus of the medium is, what terminology is used, or even at what rhythm topics are published. By combining and investigating articles of these two media types, it was possible to identify a broader range of ethical challenges that provide stimuli for questions in research and discussions regarding the Ebola epidemic: “Medical ethics can provide useful insights for decision making in epidemics, provided that you ask the right questions.” [35] In order to cope with the normative challenges of an epidemic, it is therefore important to identify underlying problems of an epidemic and their ethical implications to “[…] explore how we can improve our approach to preventing, navigating and mitigating associated ethical issues in global outbreak preparedness and response” [36].

A systematic investigation of ethical challenges can contribute to this, especially when several perspectives are taken into account. The two selected media genres in our study complement each other when it comes to identifying ethical problems of the Ebola epidemic. For example, some topics are more frequent in the lay press (e.g. regarding stigmatization or fear) or occur only there at all (e.g. resource allocation), as they follow daily reporting and respond to direct phenomena or events in the public domain. In the scientific press, the publication dynamics are different: acute phenomena are often reacted to with a time lag. Individual journal publication processes also play a role here – several weeks to months can pass between submission, review, acceptance and publication of an article.

Both the genre-specific thematic orientation of the respective media type, which is reflected in the number of articles in certain categories, and the temporally varying occurrence of topics can also be confirmed in current research literature.

For this purpose, a literature search on the PubMed database for the time period 2016-2020 was conducted in order to obtain a rough overview of research publications after the end of the Ebola epidemic that deal with aspects of medical ethics of the epidemic. The terms “Ebola” and “ethics” were used as combined keywords for the search. The resulting articles were then checked against our previously identified five categories (cf. Table 3).

**Table 3:** Distribution of the identified categories in the post-epidemic research literature (2016-2020)

	Categories	2016	2017	2018	2019	2020
1.	Responsibility and Accountability	21	4	2	-	-
2.	Spillover Effects	-	-	-	-	-
3.	Research and Development	24	9	10	6	4
4.	Health Communication	1	2	-	-	1
5.	Resource Allocation	1	-	-	-	-
	total categories	47	15	12	6	5
	total articles^a^	38	14	13	6	5

Results of the literature search on PubMed database for the years 2016-2020, ordered by number of articles (*n*)

^a^The total number listed indicates the articles considered minus those that were not accessible or that had no ethical or Ebola-specific content despite indexing

It can be seen that, on the one hand, the ethical debate on Ebola after the 2014-2016 epidemic was still present in research literature, but this presence declined significantly by 2020. On the other hand, it becomes apparent that the categories already identified in our database and the frequency distribution of these categories is also evident in research literature after the epidemic, starting 2016: the category “Research and Development” is here also the most frequently represented category as in our study. The second most common categories of scientific press in our study were “Spillover Effects” and “Responsibility and Accountability”. In the post-epidemic research literature, the category “Responsibility and Accountability” was very often represented in articles of 2016, but then declined sharply and was no longer represented at all in 2019; in this category, the above-mentioned time lag between academic journals and newspapers in responding to phenomena and thus publishing issues could have an influence: during the epidemic, this category was over-represented in lay press, while in the period after the epidemic it was also strongly present in the scientific press. “Spillover Effects” of the epidemic, such as fear or stigmatization were not discussed at all in the post-epidemic literature.

Furthermore, it can be seen that in the post-epidemic literature new subject areas have been added within the already existing categories of our study. For example, in the category “Research and Development” the reference to the topic of pregnancy and how to deal with it within a clinical study on Ebola can be found in articles from 2017 and 2019 [37–39] and in the category “Responsibility and Accountability” there are articles that focus on the topic of “care ethics” [40–42]. At the same time, a new ethical challenge in articles of 2017 was identified, which has been addressed in the post-epidemic research literature: the ethics of publication [43, 44]; here, the non-existent or limited access to data and information during the epidemic was a particular issue.

The present study has methodological limitations regarding sample size and selection bias. The qualitative results focus on the ethical challenges raised in October 2014. They thus form a partial section of the entire quantitative investigation and are characterized by the influencing factors and events of the Ebola epidemic (cf. theory of dynamics of topics within the media public). The results are reduced to a section of reality and cannot therefore be generalized in their entirety.

The academic journals and daily newspapers investigated are Western, i.e. the information and reporting comes from the Western media, which primarily has an “out-of” perspective on the Ebola epidemic rather than the direct perspective of affected regions. It would be interesting to compare debated ethical issues in the media from the affected Ebola areas with those of the present study. Furthermore, the selection of journals as well as daily newspapers was limited in terms of number and thematic focus. Here, a larger research project would provide the opportunity to include and analyze more journals and newspapers to cover a larger facet. In total, only two daily newspapers were used for the area of lay press in order to be able to process the relatively large amount of data that arises in daily reporting within the project period. Accordingly, the database contains more articles of lay than of scientific press. Another limitation concerns the number of articles that explicitly or implicitly show ethical concerns. Only 11% of the 64 articles from the two media types show explicit ethical issues, while the majority of articles (89%) show them implicitly. In order to examine this circumstance more closely, e.g. whether the media types have an influence on the presentation of ethical content, a more balanced distribution of the number of articles from the two media types would have been necessary (the number of articles for content analysis in our study included 49 from lay press and 15 from scientific press). The definition of the search unit “ethical problem” emerged as a major difficulty within the current study. There are no hard criteria within research literature regarding the formulation of what an ethical problem is or how this can be distinguished from other problems. In order to resolve this situation and to obtain the most comprehensive picture possible of ethical problems during the Ebola epidemic, a broad definition was used.

## Conclusions

The problems identified in the present study demonstrate the immense scope of ethical challenges caused by the Ebola epidemic. They range from controversial measures that deprived people of liberty to public protection, financing problems, stigmatization and misinformation to tendentious health communication.

Scientific studies investigating the Ebola epidemic from an ethical point of view focused in particular on aspects of Ebola research. The current study shows that especially the focus of academic journals remains in the area of research ethics, this is also evident in the additional search on the PubMed database for the post-epidemic period. In contrast, popular articles in newspapers focused mainly on aspects of “responsibility and accountability”.

It becomes clear that the scientific and public discourse is taking place separately and that different ethical issues of the epidemic are being considered; at the same time, they complement each other and make it possible to address the ethical challenges of this crisis more comprehensively. The results of our study represent a section of reality that cannot be generalized, but some of the ethical challenges mentioned above are also found in other outbreaks, such as the current COVID-19 pandemic, especially with regard to the ethics of research and the allocation of resources [45]. In addition, a comparative analysis of the ethical challenges raised by the HIV/AIDS pandemic and its coverage (at different points in time during the pandemic) with the media categories and types identified here should also yield informative results.

An ethical discourse on the Ebola epidemic requires an examination of as many of the ethical dimensions involved as possible. The presented investigation of the two types of media with regard to the Ebola epidemic offers this possibility of a more comprehensive insight into this diversity as a basis for ethical discussions.

## Data Availability

Data obtained in the study are available on reasonable request from the corresponding author.

## Abbreviations

WHO: World Health Organization
DRC: Democratic Republic of Congo
EVD: Ebola Virus Disease
